# Squaramide-based synthetic chloride transporters activate TFEB but block autophagic flux

**DOI:** 10.1038/s41419-019-1474-8

**Published:** 2019-03-11

**Authors:** Shaoyi Zhang, Yan Wang, Wei Xie, Ethan N. W. Howe, Nathalie Busschaert, Allan Sauvat, Marion Leduc, Lígia C. Gomes-da-Silva, Guo Chen, Isabelle Martins, Xiaxing Deng, Luigi Maiuri, Oliver Kepp, Thierry Soussi, Philip A. Gale, Naoufal Zamzami, Guido Kroemer

**Affiliations:** 1 Department of Surgery, Ruijin Hospital, Shanghai JiaoTong University School of Medicine, Shanghai, China; 2 Faculty of Medicine, University of Paris Sud-Saclay, Kremlin-Bicêtre, France; 3 Metabolomics and Cell Biology Platforms, Gustave Roussy Cancer Campus, Villejuif, France; 4 Centre de Recherche des Cordeliers, INSERM U1138 Paris, France; 5 Université Paris Descartes, Sorbonne Paris Cité, Paris, France; 6 Gustave Roussy Comprehensive Cancer Center, Villejuif, France; 7 Sorbonne Université, UPMC Univ Paris, Paris, France; 8 Department of Radiation Oncology, Fudan University Shanghai Cancer Center, Shanghai, China; 9 School of Chemistry, The University of Sydney, Sydney, NSW 2006 Australia; 10 Chemistry Department, University of Southampton, Southampton, UK; 11 Chemistry Department, University of Coimbra, Coimbra, Portugal; 12 European Institute for Research in Cystic Fibrosis, Division of Genetics and Cell Biology, San Raffaele Scientific Institute, Milan, Italy; 13 Department of Health Sciences, University of Piemonte Orientale, Novara, Italy; 14 Department of Oncology-Pathology, Cancer Center Karolinska (CCK), Karolinska Institutet, Stockholm, Sweden; 15 Pôle de Biologie, Hôpital Européen Georges Pompidou, APsupp-HP, Paris, France; 16 Department of Women’s and Children’s Health, Karolinska University Hospital, Stockholm, Sweden

## Abstract

Cystic fibrosis is a disease caused by defective function of a chloride channel coupled to a blockade of autophagic flux. It has been proposed to use synthetic chloride transporters as pharmacological agents to compensate insufficient chloride fluxes. Here, we report that such chloride anionophores block autophagic flux in spite of the fact that they activate the pro-autophagic transcription factor EB (TFEB) coupled to the inhibition of the autophagy-suppressive mTORC1 kinase activity. Two synthetic chloride transporters (**SQ1** and **SQ2**) caused a partially TFEB-dependent relocation of the autophagic marker LC3 to the Golgi apparatus. Inhibition of TFEB activation using a calcium chelator or calcineurin inhibitors reduced the formation of LC3 puncta in cells, yet did not affect the cytotoxic action of **SQ1** and **SQ2** that could be observed after prolonged incubation. In conclusion, the squaramide-based synthetic chloride transporters studied in this work (which can also dissipate pH gradients) are probably not appropriate for the treatment of cystic fibrosis yet might be used for other indications such as cancer.

## Introduction

Cystic fibrosis is the most frequent monogenetic lethal disease affecting humans^1,2^. This pathology is caused by loss-of-function mutation in the cystic fibrosis transmembrane conductance regulator (CFTR), a chloride transporter^3,4^. Importantly, CFTR mutations do not only compromise chloride flux (resulting in increased chloride concentrations in sweat and alterations in the composition of mucus with subsequent bronchial infections) but also affect autophagic flux in respiratory epithelial cells and macrophages^5–10^. The perturbation of autophagy may be disease-relevant because autophagy induction by pharmacological inhibitors of E1A-associated protein p300 (where E1A = adenovirus early region 1A), best known as EP300, and transglutaminase-2 can favor the function of certain CFTR mutants (and in particular the del508 mutant protein)^11–14^ and reduce lung inflammation in patients^15–17^.

Intrigued by these observations, we wondered whether direct perturbations of chloride homeostasis by means of synthetic chloride transporters (or anionophores) such as the squaramide-based compound **SQ1** and the analogous **SQ2**^18,19^ might affect the autophagic process. **SQ1** and **SQ2** incorporate into the plasma membrane (and perhaps other cellular membranes), thus causing chloride influx into cells and dissipating chloride gradients in intracellular compartments, and neutralizing lysosomal pH in the process^19^. Although this ultimately causes cell death (and **SQ1** has indeed been developed as a first-in-class anticancer agent), **SQ1** and **SQ2** can modulate autophagy before cells die^19^, offering a window of opportunity for investigating their impact on the system.

Here, we report that squaramide-based synthetic chloride transporters dramatically inhibit autophagic flux although they activate a pro-autophagic transcription factor. Moreover, these chloride anionophores trigger the relocation of an autophagic marker (LC3) towards the Golgi apparatus.

## Materials and methods

### Cell culture and transfection

Human Osteosarcoma U2OS cells were cultured at 37 °C and 5% CO_2_ in Dulbecco’s modified Eagle’s medium (DMEM; Life Technologies) supplemented with 100 mM 2-[4-(2-hydroxyethyl) piperazin-1-yl] ethanesulfonic acid (HEPES) buffer, 10% heat-inactivated fetal bovine serum (FBS) (Life Technologies) and 1% penicillin/streptomycin (Life Technologies).

GFP-LC3 stable cell lines were generated by transducing U2OS WT, U2OS ATG5^−/−^, TFEB^−/−^, CFBE Delta-F508 with pre-packaged viral particles expressing recombinant GFP-LC3 (LentiBrite GFP-LC3B Lentiviral Biosensor; Millipore, 17–10193). U2OS cells stably expressing GFP-TFEB were transfected with the pEGFP-N1-TFEB plasmid using the FuGENE® HD transfection reagent protocol. The pEGFP-N1-TFEB plasmid was a gift from Shawn Ferguson (Addgene plasmid # 38119). Subsequently, stable expressing cells were selected by means of appropriate selection antibiotics and clones were obtained by single cell sorting using a FACS DIVA (Becton Dickinson, Franklin Lakes, NJ, USA). U2OS GALT-GFP LC3-RFP cells were constructed with the protocol as published in our pervious paper^20^ with a plasmid coding for GALT1(β1,4-galactosyltransferase 1)-GFP, and then transfected with lentiviral particles coding for RFP-LC3 from Merck Millipore(17–10143). PC12 GFP-Q74 cells are generous gift from David Rubinsztein’s lab from Cambridge^21^.

### Chemicals and antibodies

The squaramide compound **SQ1**, its analogue **SQ2**, and the control compound **SQ3** (with no chloride transport activity) were previously described^18,19^. The following chemicals and antibodies were used in this study: Torin 1, bafilomycin A1, BAPTA AM, Cyclosporin A (Tocris Bioscience, Bristol, UK), Cycloheximide, Cyclosporin H, N-Acetyl-L-cysteine, L-Glutathione reduced (Sigma-Aldrich) oleate from Larodan (Solna, Sweden).

Anti-beta actin (Abcam 8226, Cambridge, UK), LC3 (#3868), mTOR (#2983), Phospho-mTOR (Ser2448) (#5536), LAMP1 (#9091), p70 S6 Kinase (#9202), Phospho-p70 S6 Kinase (Thr389) (#9205), SQSTM1/p62 (#7695) antibody were all purchased from Cell Signaling Technology, IF antibody Alexa Fluor® 647 anti-human CD107a (LAMP-1) Antibody from BioLegend.

### Cytofluorometry measurement of cell death

Cell viability was evaluated by co-staining the cells, during 30 min at 37 °C, with 40 nM 3,3′dihexiloxalocarbocyanine iodide (DiOC6(3)), Molecular Probes/Invitrogen), a mitochondrial transmembrane potential sensitive dye, and 2 μM DAPI (all from Molecular Probes-Life Technologies, Carlsbad, CA, USA). Cytofluorometric acquisitions were carried out on a Milteny cytofluorometer (MACSQuant® Analyzer 10), and statistical analyses were performed by using the FlowJo software (LLC, Oregon, USA).

### Cytofluorometry measurement of ROS generation

The generation of ROS was monitored with hydroethidine (HE) (at a final concentration of 5 μM; stock 10 mM in DMSO; excitation wave length of 488 nM, emission 620 nM; Molecular Probe. Briefly, cells were collected after treatment with the testing compounds followed by co-staining, during 30 min at 37 °C, with HE. Cytofluorometric acquisitions were carried out on a Milteny cytofluorometer (MACSQuant® Analyzer 10), and statistical analyses were performed by using the FlowJo software (LLC, Oregon, USA).

### High content image acquisition

Cells were seeded in tissue culture-treated 384-well μClear imaging plates (Greiner BioOne, Frickenhausen, Germany) and incubated under standard tissue culture conditions during 24 h at 37 °C. Then, cells were treated with the indicated compounds and after 6 or 24 h of incubation, cells were fixed with 4% formaldehyde solution containing 1 μM Hoechst 33342 overnight at 4 °C. The fixative was changed to PBS, and the plates were subjected to automated image analysis. For automated fluorescence microscopy, a robot-assisted Molecular Devices IXM XL BioImager (Molecular Devices, Sunnyvale, CA, USA) equipped with Sola light sources (Lumencor, Beaverton, OR, USA), adequate excitation and emission filters (Semrock, Rochester, NY, USA), and a 16-bit monochromes sCMOS PCO.edge 5.5 camera (PCO, Kelheim, Germany) and a ×20 PlanAPO objective (Nikon, Tokyo, Japan) was used to acquire nine view fields/well, followed by image processing with the custom module editor of the MetaXpress software (Molecular Devices). For the latter, the images were segmented and analyzed for GFP, RFP granularity or global fluorescence intensity (depending on the dyes) by comparing the standard deviation of the mean fluorescence intensity of groups of adjacent pixels within the cytoplasm of each cell to the mean fluorescence intensity in the same ROI using the MetaXpress software (Molecular Devices).

### Immunoblotting

Immunoblotting was performed following standard procedures. Cells were harvested and the obtained pellet was resuspended in RIPA buffer (89900; Thermo Fisher Scientific) supplemented with phosphatase and protease inhibitors (88669; Thermo Fisher Scientific) followed by sonication and protein content quantification by DCTM Protein Assay kit (5000112; Bio-Rad, Hercules, CA, USA). Then, 10 μg of protein were separated on NuPAGE Novex Bis-Tris 4–12% pre-cast gels (Invitrogen-Life Technologies, Carlsbad, CA, USA) and transferred to Immobilon polyvinylidene difluoride membranes (Merck-Millipore, Darmstadt, Germany). Unspecific binding was reduced by incubating the membranes for 1 h in 0.05% Tween 20 (v/v in TBS) supplemented with 5% w/v bovine serum albumin (Euromedex, Souffelweyersheim, France). Following, proteins were probed with antibodies specific for actin, LC3, mTOR, Phospho-mTOR (Ser2448), LAMP1, p70 S6 Kinase or SQSTM1/p62. Primary antibodies were revealed with species-specific immunoglobulin G conjugated to horseradish peroxidase (Southern Biotech, Birmingham, AL, USA), followed by chemiluminescence analysis with the SuperSignal West Pico reagent by means of an ImageQuant 4000 (GE Healthcare, Little Chalfont, UK).

### Statistical analyses

Data are reported as means ± SD of *n* > 3 replicates and experiments were repeated at least twice yielding similar results. Data were analyzed using Prism (GraphPad Software, Inc., La Jolla, CA, USA), and statistical significance was assessed by means of two-tailed Student’s *t*-test or ANOVA tests, as appropriate. Unless otherwise specified, data are reported as mean ± SEM. Statistical significance was analyzed using the Student’s-test. Differences in treated and control cells were considered to be significant if *p < 0.05, **p < 0.01, ***p < 0.001.

## Results

### SQ1 and SQ2 induce autophagic LC3^+^ puncta in U2OS cells

**Fig. 1:**
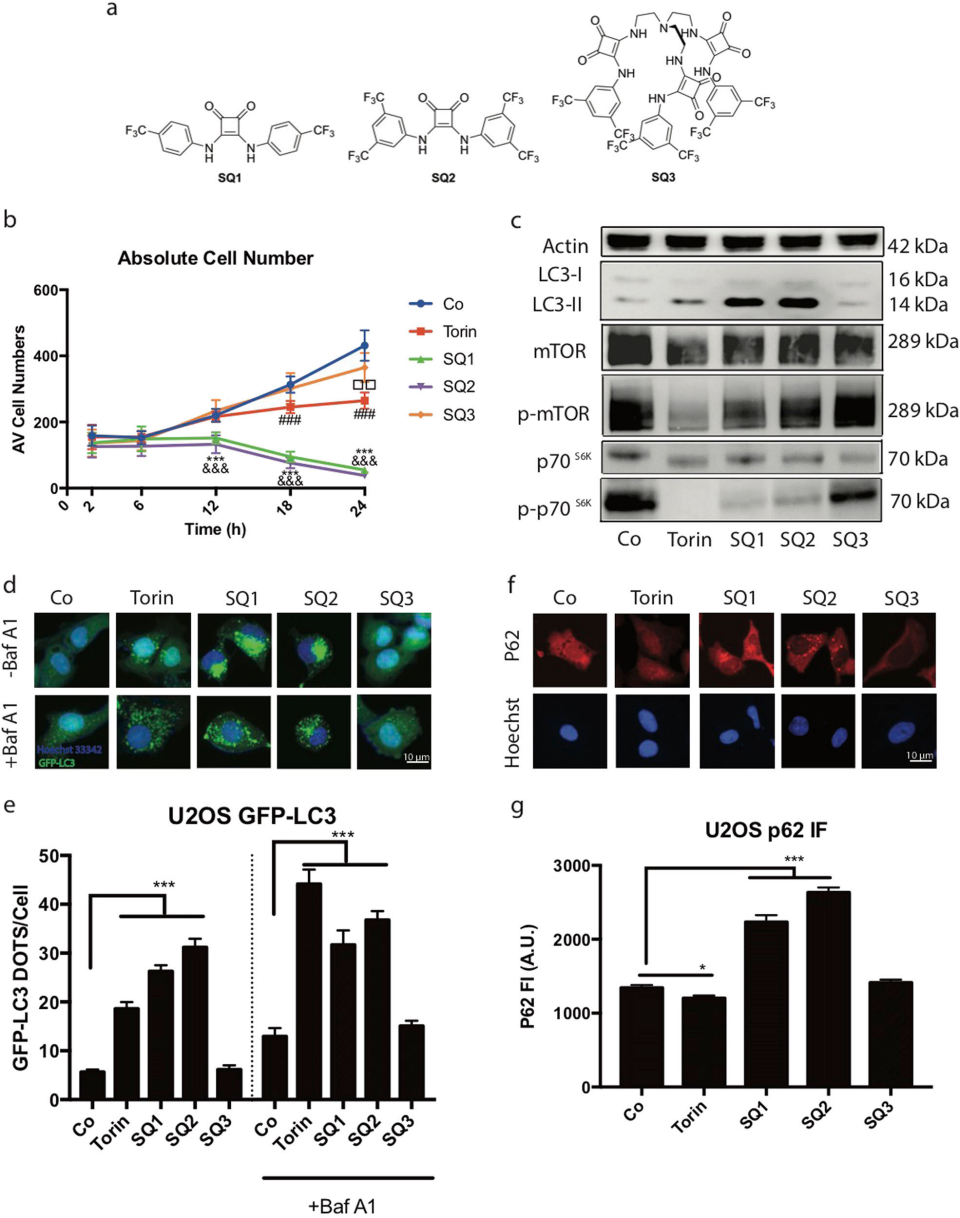
Chemical structure of SQ1 and SQ2 and their effect on autophagic parameters. **a** Structure of squaramide-based synthetic chloride transporters **SQ1**, **SQ2**, and **SQ3**. **b** Kinetic studies of Torin 300 nM, **SQ1**, **SQ2**, and **SQ3** at 10 μM on U2OS human osteosarcoma cells from 2–24 h. Cell numbers were determined by means of fluorescence microscopy upon Hoechst staining. The graph depicts the average cell number for each treatment per site of acquisition in a 384 well plate. **c** Immunoblot analysis of LC3 lipidation, protein expression level of mTOR, p70^S6K^ and their phosphorylated forms(Thr389) after 6-hour treatments. **d**, **e** Representative images and statistical analysis of U2OS GFP-LC3 cells after treatment with Torin 300 nM or **SQ1**, **SQ2**, and **SQ3** at 10 μM during 6 h having bafilomycin A1(100 nM) presented in the last 2 h of treatment. **f**, **g** Representative images and statistical analysis of U2OS submitted to p62 immunofluorescence staining after 6 h of treatment with torin (300 nM) or **SQ1**, **SQ2**, and **SQ3** (10 μM). The bar chart indicated the global fluorescence intensity of p62 per cell, followed by p62 immunofluorescence staining. Data are expressed as means ± SEM of at least three independent experiments (***/&&&/###p < 0.0001, compared to untreated cells, Co)

**Fig. 2:**
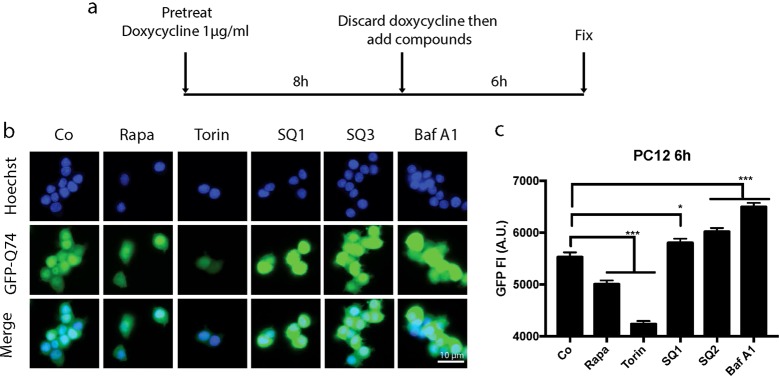
Effects of SQ1 and SQ2 on autophagic flux. **a** Scheme of the design of the experiment. **b** Representative images of PC12 GFP-Q74 cell after treatment with rapamycin (Rapa, 10 μM), torin 300 nM and **SQ1**, **SQ2**, **SQ3** respectively at 10 μM for 6 h. **c** Data are expressed as means ± SD of one representative experiment and represent the global GFP fluorescent intensity per cell. (*p < 0.05, ***p < 0.0001, compared to untreated cells, Co)

Our laboratory has made extensive use of U2OS biosensor cell lines to measure cellular stress responses including autophagy^12,22–28^. We first determined the kinetics of potential cytotoxic effects of the synthetic chloride channels **SQ1** and **SQ2** on these cells to study autophagy in conditions in which U2OS cells fully conserve their viability. These measurements led to the conclusion that the **SQ1** and **SQ2** have cytostatic effects that become significant at 12 h of incubation and diminishes the number of cells below the control value, indicating cytotoxicity, from 18 h. However, at 6 h neither cytostatic nor cytotoxic effects were detectable (Fig. 1b). Hence, all subsequent experiments were done in this time frame (6 h). **SQ1** and **SQ2** both induced the lipidation of microtubule-associated proteins 1A/1B light chain 3B (hereafter referred to as LC3 II), as detectable by an increase in the electrophoretic mobility of LC3 (Fig. 1c). Moreover, both synthetic chloride anionophores (but not the control compound **SQ3**, which shares structural features with **SQ2**, yet lacks chloride transporter activity^19^) inhibited the kinase activity of mechanistic target of rapamycin complex-1 (mTORC1), as indicated by a reduced phosphorylation of ribosomal protein S6 kinase beta-1 (S6K1, best known as p70S6K) (Fig. 1c). Commensurate with these effects, **SQ1** and **SQ2** (but not **SQ3**) stimulated the aggregation of green fluorescent protein (GFP)-LC3 fusion protein in cytoplasmic dots (Fig. 1d). This effect was found both in the absence and in the presence of bafilomycin A1 (BafA1), an inhibitor of the lysosomal vATPase (Fig. 1e). However, **SQ1** and **SQ2** did not induce autophagic flux, as indicated by two independent series of experiments. First, in the presence of **SQ1** and **SQ2**, the abundance of the autophagic substrate sequestosome 1 (STQM1, best known as p62) in U2OS cells did not decrease (as this was observed with the positive control, torin, an inhibitor of mTORC1 and mTORC2) but increased (Fig. 1f, g). This increase probably reflects the impairment of lysosomal function due to the decrease of the activity of the pH-sensitive hydrolases. This loss of lysosomal function is due to abnormal pH increase in lysosomes when intracellular chloride concentration is dysregulated. Second, in the neuronal PC12 cell line engineered to express a doxycyclin-inducible autophagic cargo (namely a GFP fused to exon 1 of a pathogenic huntingtin protein variant that contains 74 glutamine repeats, Q74)^29^, **SQ1** and **SQ2** increased the level of the cargo, contrasting with the effects of torin or rapamycin that both caused its elimination (Fig. 2).

In sum, **SQ1** and **SQ2** stimulate LC3 lipidation, its redistribution towards puncta, yet do not stimulate autophagic flux, confirming previous observations made in HeLa cells^19^.

### Golgi localization of LC3 after SQ1/SQ2 treatment

**Fig. 3:**
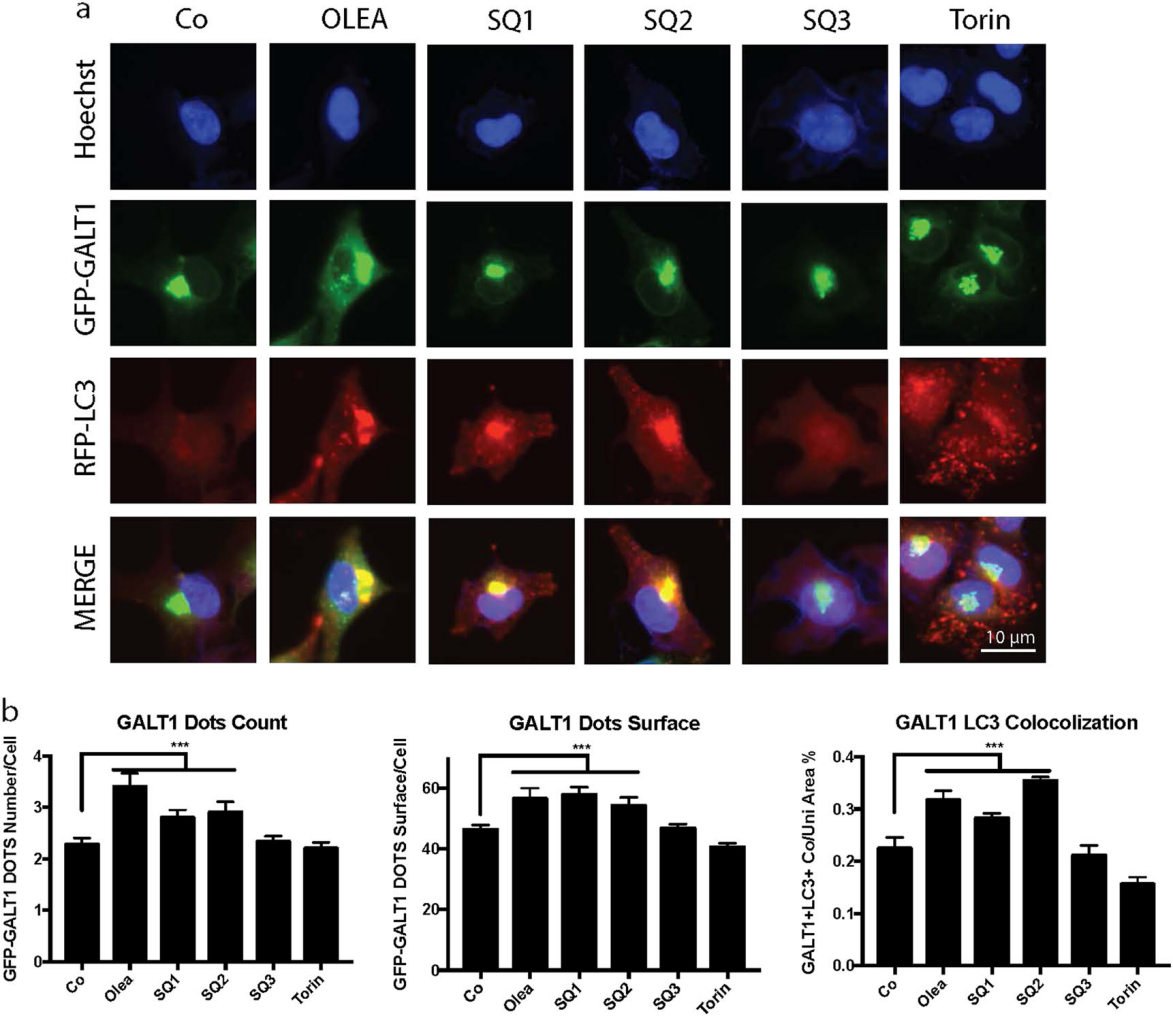
SQ1and SQ2 induced Golgi localization of LC3. Representative images and statistical analysis of U2OS cells expressing GFP-GALT RFP-LC3 cells treated with torin (300 nM), oleate (500 μM), **SQ1**, **SQ2** or **SQ3** (10 μM) for 6 h. Quantitative analysis represents the number and area of GALT1^+^ Golgi structures per cell and the colocalization of LC3^+^ and GALT1^+^ structures. Data are expressed as means ± SD of one representative experiment (***p < 0.0001, compared to untreated cells, Co). Representatie images are showin in panel **a** and quantitative comparisions are detailled in panel **b**

**Fig. 4:**
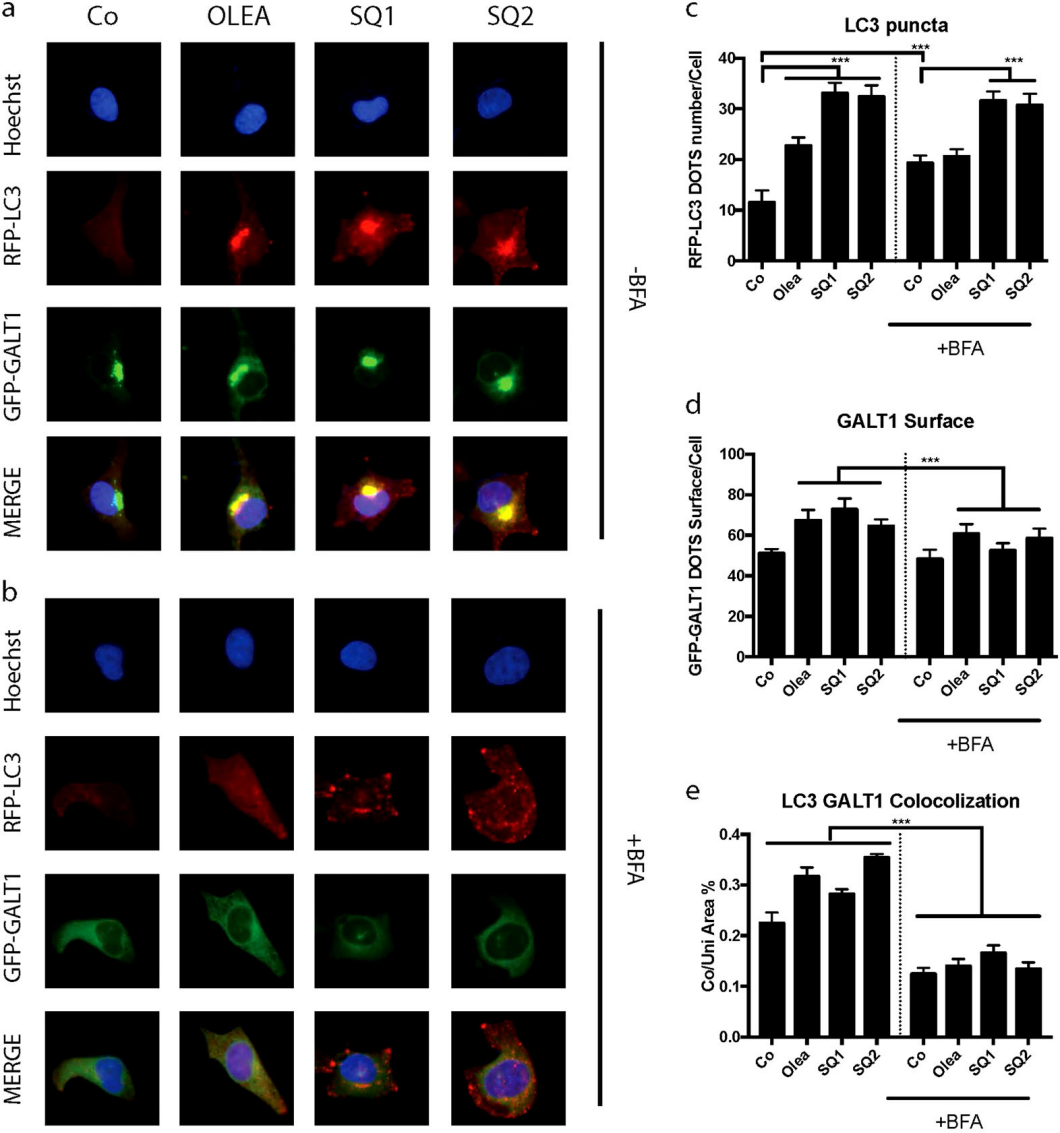
Brefeldin A reduced SQ1 and SQ2 induced translocation of LC3 to the Golgi. **a**, **b** Representative images of U2OS GFP-GALT RFP-LC3 cells treated with the compounds for 6 h (oleate 500 μM, **SQ1**, **SQ2** at 10 μM) without (**a**) or after pretreatment with brefeldinA (10 μg/ml) for 1 h (**b**). **c**–**e** Statistical analysis of RFP-LC3 puncta counts per cell, GFP-GALT marked Golgi surface per cell and the localization of LC3 and GALT (***p < 0.0001, compared to untreated control cells, Co)

The GFP-LC3 puncta induced by **SQ1** or **SQ2** were larger than those induced by torin and tended to coalesce at one pole of the nucleus (Fig. 1). We therefore suspected that such puncta might aggregate at or close to the Golgi apparatus. To examine this hypothesis, we took advantage of a U2OS biosensor cell line expressing a red fluorescent protein (RFP)-LC3 fusion protein as a reporter of LC3 localization, as well as a GFP-galactose-1-phosphate uridylyltransferase (GALT) fusion protein as a reporter of Golgi location^20,22,24,30,31^. After treatment with **SQ1** or **SQ2**, we found a remarkable overlap of both fluorescent signals indicating the translocation of LC3 to the Golgi, a phenotype that closely resembled the one induced by oleate^20,25^ but differed from that induced by torin (Fig. 3). To further investigate this phenomenon, we pretreated the cells for 1 h with brefeldin A (BFA), an inhibitor of GBF1 (brefeldin A-resistance guanine nucleotide exchange factor 1) that disrupts the Golgi apparatus causing its dispersion into ministacks all over the cytoplasm^32–34^. Preincubation with BFA reduced the colocalization of GFP-GALT and RFP-LC3, confirming the idea that LC3 coalesces at the Golgi, yet did not prevent the **SQ1**/**SQ2**-induced formation of RFP-LC3 dots, suggesting that the Golgi is not required for this phenomenon to occur (Fig. 4).

### Contribution of TFEB to LC3 relocation

**Fig. 5:**
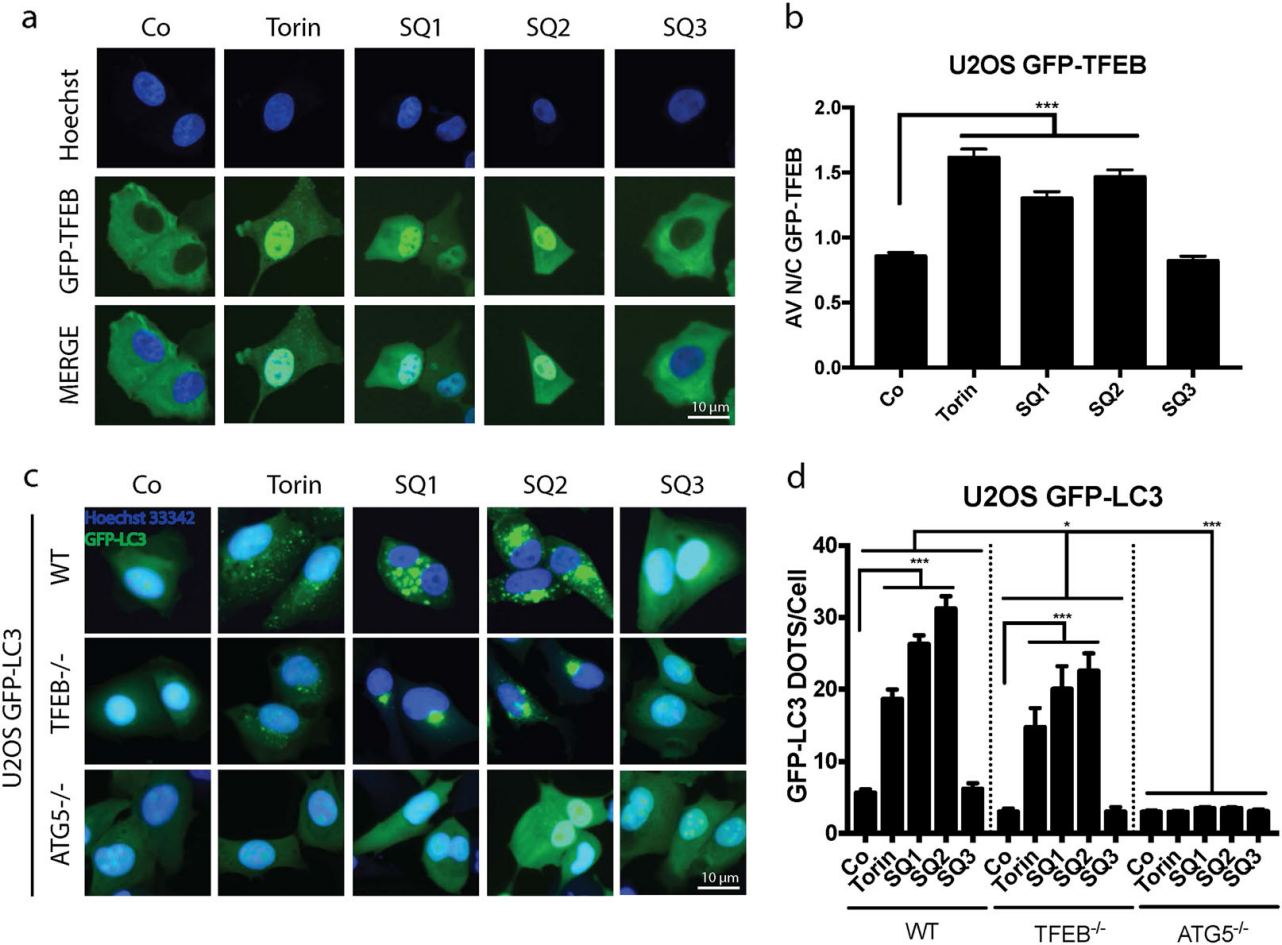
Contribution of TFEB and ATG5 in LC3 relocation induced by the SQs. **a**, **b** Representative images of U2OS cells stably expressing GFP-TFEB fusion protein, treated with torin 300 nM, **SQ1**, **SQ2**, and **SQ3** at 10 μM during 6 h. Data are expressed as means ± SEM of at least three independent experiments and demonstrate the average ratio between GFP-TFEB florescence intensity in the nucleus vs. cytoplasm. (*p < 0.05, **p < 0.01, ***p < 0.0001, compared to untreated cells, Co). **c**, **d** Representative images and statistics of U2OS GFP-LC3 cells, and the ATG5 KO and TFEB KO counterparts after 6 h of treatment with the mentioned compounds. Bars depicts the absolute number of GFP-LC3^+^ dots per cell. Data are expressed as means ± SEM of at least three independent experiments (*p < 0.05, ***p < 0.0001, compared to untreated cells, Co)

Since **SQ1** and **SQ2** inhibit mTORC1, and this inhibition has been involved in the activation of the pro-autophagic transcription factor EB (TFEB)^35–39^, we investigated whether these synthetic chloride transporters would cause the translocation of a GFP-TFEB reporter protein from the cytoplasm to the nucleus. Indeed, **SQ1** and **SQ2** (but not **SQ3**) were able to stimulate TFEB activation (Fig. 5a, b). TFEB is known to stimulate lysosomal biogenesis, and both **SQ1** and **SQ2** caused the overexpression of lysosomal-associated membrane protein 1 (LAMP-1) in U2OS cells (Fig. S1). Moreover, knockout of *TFEB* partially reduced the formation of LC3B puncta induced by **SQ1** or **SQ2**, an inhibition that was far less prominent than that found for the knockout of *ATG5* (Fig. 5c, d). Quantitation of GFP-LC3 puncta that co-stain with an antibody recognizing LAMP1 revealed that SQ1 and SQ2 did not inhibit the fusion between lysosomes and autophagosomes (Fig. S3e). These observations suggest that SQ compounds inhibit autophagic flux at a step downstream of the autophagosome-lysosome fusion, presumably because SQ1 and SQ2 dissipate the (normally acidic) pH gradient on the lysosomal membrane^19^.

**Fig. 6:**
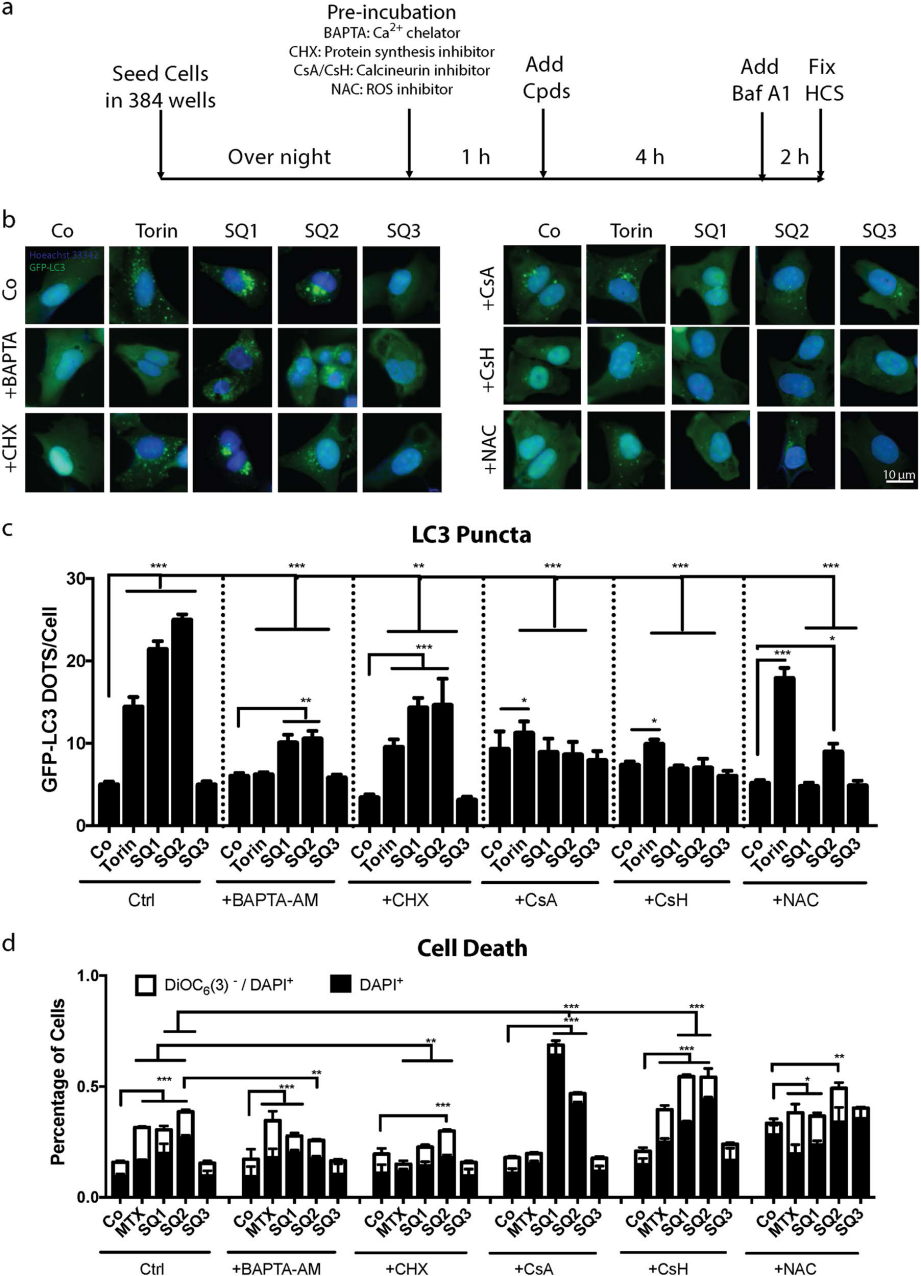
Effects of pharmacological inhibition of TFEB on LC3 relocation and cell death induced by SQ1 and SQ2. **a** Experimental design to assess LC3 puncta when the synthetic chloride transporters **SQs** were combined with different inhibitors. **b** Representative images of U2OS GFP-LC3 expressing cells when pretreated with the calcium chelator BAPTA-AM, the antioxydant NAC, the calcineurin inhibitor CsA, its analogue CsH, and the protein synthesis inhibitor CHX followed by the co-treatment with the synthetic chloride modulators. **c** Bars show the average number of GFP-LC3^+^ dots per cell. Data are expressed as means ± SD of one representative experiment *P < 0.05; **P < 0.01; ***P < 0.001. **d** Evaluation of cell death by double-staining with DAPI together with DiOC_6_(3) followed by cytofluorometric detection of dead cells (DAPI^+^) and dying cells (DAPI^+^ DiOC_6_(3)^low^). Data are expressed as means ± SEM of at least three independent experiments (Co/Ctrl). *P < 0.05; **P < 0.01; ***P < 0.001

Intrigued by these observations, we decided to investigate the role of TFEB in the induction of LC3B puncta by inhibiting calcineurin, the phosphatase that dephosphorylates TFEB for its activation after mTORC1 inhibition^35,40,41^. For this, we either used the calcium chelator 2,2′-(ethylenedioxy)dianiline-N,N,N,N-tetraacetic acid (BAPTA) that can be targeted into cells a cell-permeant acetoxymethyl ester derivative (BAPTA-AM) or, more specifically, enzymatic inhibitors of calcineurin such as cyclosporine A (CsA) and its non-immunosuppressive control compound cyclosporin H (CsH). Moreover, we used cycloheximide (CHX) as a general inhibitor of protein translation. Cells were preincubated for 1 h with these reagents and then were treated with **SQ1** or **SQ2**. Of note, BAPTA-AM, CsA, and CsH strongly inhibited the formation of GFP-LC3 puncta (Fig. 6b, c). CsA and CsH failed to inhibit the formation of reactive oxygen species (ROS) induced by **SQ1** and **SQ2** (as measurable by quantifying the conversion of non-fluorescent hydroethidine into fluorescent ethidium), which however could be quenched by using the antioxidant N-acetyl cysteine (NAC) (Fig. S2). NAC was also capable of inhibiting LC3 puncta formation (Fig. 6b, c). Importantly, none of the inhibitors of GFP-LC3 puncta (i.e., BAPTA-AM, CHX, CsA, CsH, NAC) nor full inhibition of the autophagic process (in ATG5 KO cells) (Fig. S3C) were able to significantly reduce cell killing by **SQ1** or **SQ2**, as measured at 24 h of incubation. Hence, the formation of GFP-LC3 puncta and later cell death can be uncoupled from each other.

Furthermore, to evaluate the possibility that CsA and TFEB act on different steps of the cellular alterations induced by **SQ1** and **SQ2**, we compared the SQ1 and SQ2 effects on the formation of GFP-LC3 dots between U2OS WT and U2OS TFEB KO cells in the presence or absence CsA. The CsA-mediated inhibition SQ1 and SQ2-elicited GFP-LC3 dots was maintained in TFEB KO cells. This argues in favor of the possibility that TFEB and CsA modulate the effects of **SQ1** and **SQ2** in a differential fashion (Fig. S3a, b).

Finally, we treated a cystic fibrosis bronchial epithelial (CFBE) cell line harboring the most frequent CFTR mutation (delF508) for 24 h with **SQ1** or **SQ2** in the absence or presence of bafilomycin A1. The results indicate that **SQ1** or **SQ2** induced the formation of GFP-LC3 dots and that this effect was not further enhanced by bafilomycin A1 (Fig. S3F), confirming that **SQ1** or **SQ2** inhibit autophagic flux in a disease-relevant cellular model.

## Discussion

**SQ1** and **SQ2** have previously been shown to stimulate the lipidation of LC3^19^. Here, we confirm this finding and link it to the relocation of LC3 to discrete structure in the cytoplasm, which occurs in a strictly ATG5-dependent fashion. We found that **SQ1** and **SQ2** inhibit mTORC1, a phenomenon that is linked to the activation of TFEB (and other similar transcripition factors)^19^. Indeed, **SQ1** and **SQ2** promote the translocation of TFEB from the cytoplasm to the nucleus, and TFEB is partially required for the aggregation of LC3 in discrete cytoplasmic puncta. Although TFEB activation should stimulate autophagic flux, we found that **SQ1** and **SQ2** were unable to do so. Rather both **SQ1** and **SQ2** caused the accumulation of autophagic cargo in two different cell types, in human osteosarcoma U2OS cells (in which SQSTM1 became more abundant) and in rat neuronal PC12 cells (in which a mutant Huntingtin Q74 construct accumulated). **SQ1** and **SQ2** are synthetic chloride transporters that dissipate the lysosomal pH gradient, a phenomenon that previously has been suggested to account for the inhibition of autophagic flux^19^. Thus, although both agents activate a pro-autophagic transcription factor they ultimately fail to stimulate autophagic flux.

Several strategies designed to inhibit the calcineurin-mediated activation of TFEB, namely intracellular calcium chelation or direct calcineurin inhibition by CsA largely prevented autophagy induction by **SQ1** or **SQ2**, confirming the importance of this pathway for the observed phenotype. However, inhibition of protein neo-synthesis or the calcineurin pathway was unable to prevent **SQ1** and **SQ2**-induced cell death in conditions in which such an inhibition fully suppressed the formation of LC3 puncta. We conclude from these results that the generation of such puncta apparently is not required for the cytotoxic action of synthetic chloride transporters, which must rely on other damage pathways to kill cells.

Surprisingly, **SQ1** and **SQ2** caused the relocation of LC3 into discrete perinuclear structures, usually presenting as one single cap in immediate vicinity of the nucleus. These structures turned out to co-localize with the Golgi apparatus. We previously found a similar Golgi-specific coalescence of LC3 in cells treated with unsaturated fatty acids (such as oleate)^20,42^, the photosensitizer redaporfin^22,43^, and the cytolytic peptide derivative LTX-401, which itself has a strong tropism for the Golgi^25^. Thus, structurally rather distinct compounds can induce a similar coalescence of LC3 in or at the Golgi apparatus. For all these compounds (**SQ1**, **SQ2**, oleate, redaporfin, LTX401), the Golgi-disrupting agent BFA prevented the relocalization of LC3 to perinuclear caps. The mechanisms explaining this organelle-specific pattern of LC3 redistribution remain to be elucidated.

Synthetic chloride transporters have been developed as hypothetical remedies against cystic fibrosis^44–49^. Indeed, at least theoretically, such ionophores might be used as substitutes to compensate for the defective function of CFTR^44,50^. As shown in this paper, consistent with precedent reports^19,51^, chloride ionophores that can dissipate pH gradients, such as the compounds studied here that have been shown to be capable of transporting both chloride and protons across lipid bilayers, block autophagic flux in a peculiar manner, arguing against their therapeutic utility. Indeed, it appears to be advantageous to restore autophagic turnover in cystic fibrosis^12,15,17^, implying that autophagy inhibition would ultimately worsen the disease. These types of transporter should be exploited for therapeutic use against other diseases, for instance cancer, in which their cytotoxic potential might be advantageous. The development of chloride transporters which do not dissipate pH gradients is ongoing in Sydney^52^.

## Supplementary information

Figure S1

Figure S2

Figure S3

Supplementary figure legends
