# Author Correction: Squaramide-based synthetic chloride transporters activate TFEB but block autophagic flux

**DOI:** 10.1038/s41419-019-1536-y

**Published:** 2019-04-03

**Authors:** Shaoyi Zhang, Yan Wang, Wei Xie, Ethan N. W. Howe, Nathalie Busschaert, Allan Sauvat, Marion Leduc, Lígia C. Gomes-da-Silva, Guo Chen, Isabelle Martins, Xiaxing Deng, Luigi Maiuri, Oliver Kepp, Thierry Soussi, Philip A. Gale, Naoufal Zamzami, Guido Kroemer

**Affiliations:** 1 Department of Surgery, Ruijin Hospital, Shanghai JiaoTong University School of Medicine, Shanghai, China; 2 Faculty of Medicine, University of Paris Sud-Saclay, Kremlin-Bicêtre, France; 3 Metabolomics and Cell Biology Platforms, Gustave Roussy Cancer Campus, Villejuif, France; 4 Centre de Recherche des Cordeliers, INSERM U1138 Paris, France; 5 Université Paris Descartes, Sorbonne Paris Cité, Paris, France; 6 Gustave Roussy Comprehensive Cancer Center, Villejuif, France; 7 Sorbonne Université, UPMC Univ Paris, Paris, France; 8 Department of Radiation Oncology, Fudan University Shanghai Cancer Center, Shanghai, China; 9 School of Chemistry, The University of Sydney, Sydney, NSW 2006 Australia; 10 Chemistry Department, University of Southampton, Southampton, UK; 11 Chemistry Department, University of Coimbra, Coimbra, Portugal; 12 European Institute for Research in Cystic Fibrosis, Division of Genetics and Cell Biology, San Raffaele Scientific Institute, Milan, Italy; 13 Department of Health Sciences, University of Piemonte Orientale, Novara, Italy; 14 Department of Oncology-Pathology, Cancer Center Karolinska (CCK), Karolinska Institutet, Stockholm, Sweden; 15 Pôle de Biologie, Hôpital Européen Georges Pompidou, APsupp-HP, Paris, France; 16 Department of Women’s and Children’s Health, Karolinska University Hospital, Stockholm, Sweden

**Correction to: Cell Death & Disease**

10.1038/s41419-019-1474-8

published online 11 March 2019

In the version of this article originally submitted, it was stated that the first three authors (Shaoyi Than, Yan Wang, Wei Xie) had contributed equally. However, in the published version this information was missing.

This has been corrected in both the PDF and HTML versions of the Article.

