# Supplementary figures and images for: Squaramide-based synthetic chloride transporters activate TFEB but block autophagic flux
Source: Cell Death Dis. 2019 Mar 11;10(3):242. doi: 10.1038/s41419-019-1474-8 (PMC6411943; doi:10.1038/s41419-019-1474-8)

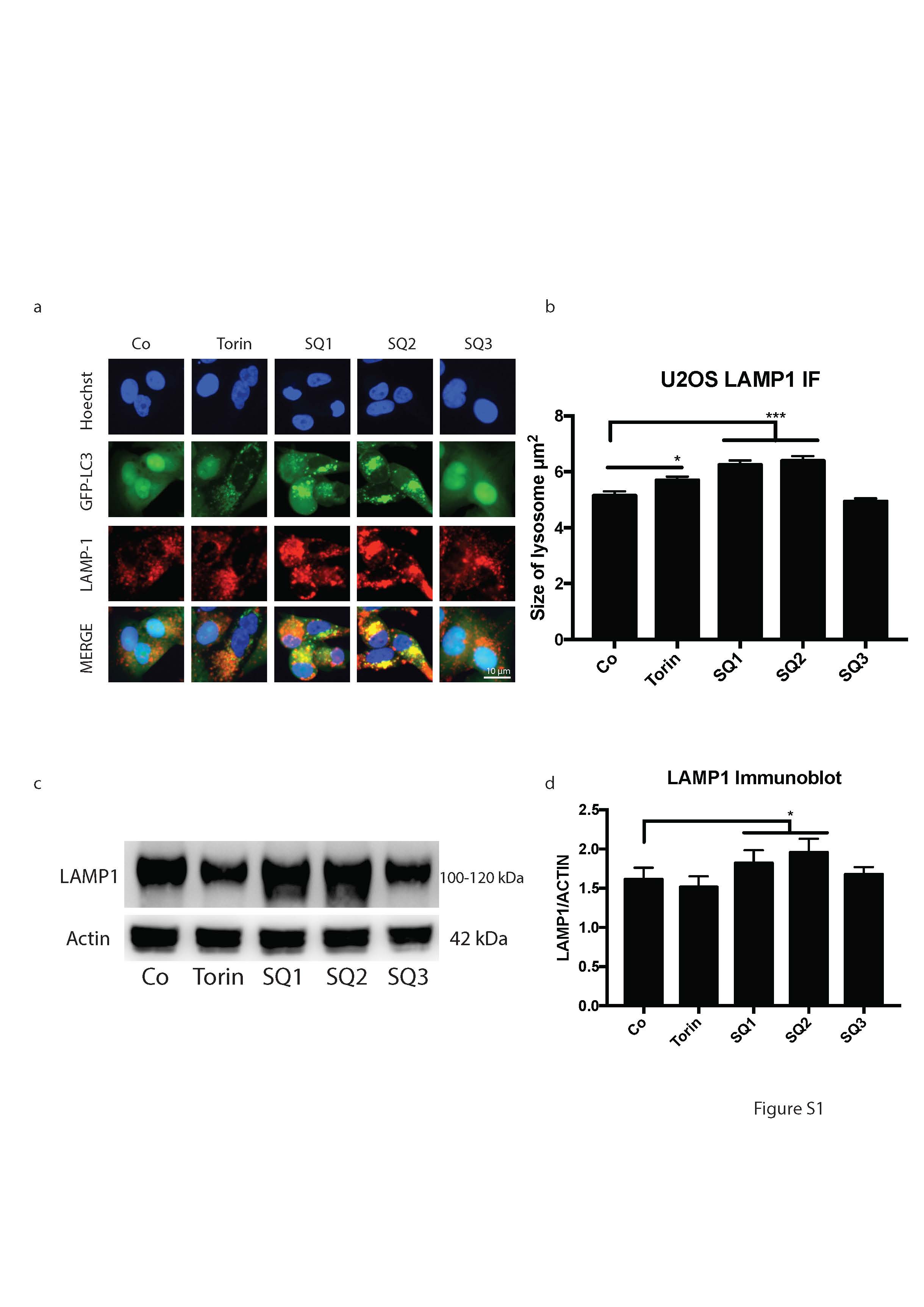

Supplement: Supplementary file 1 — Figure S1 [file 41419_2019_1474_MOESM1_ESM.jpg]

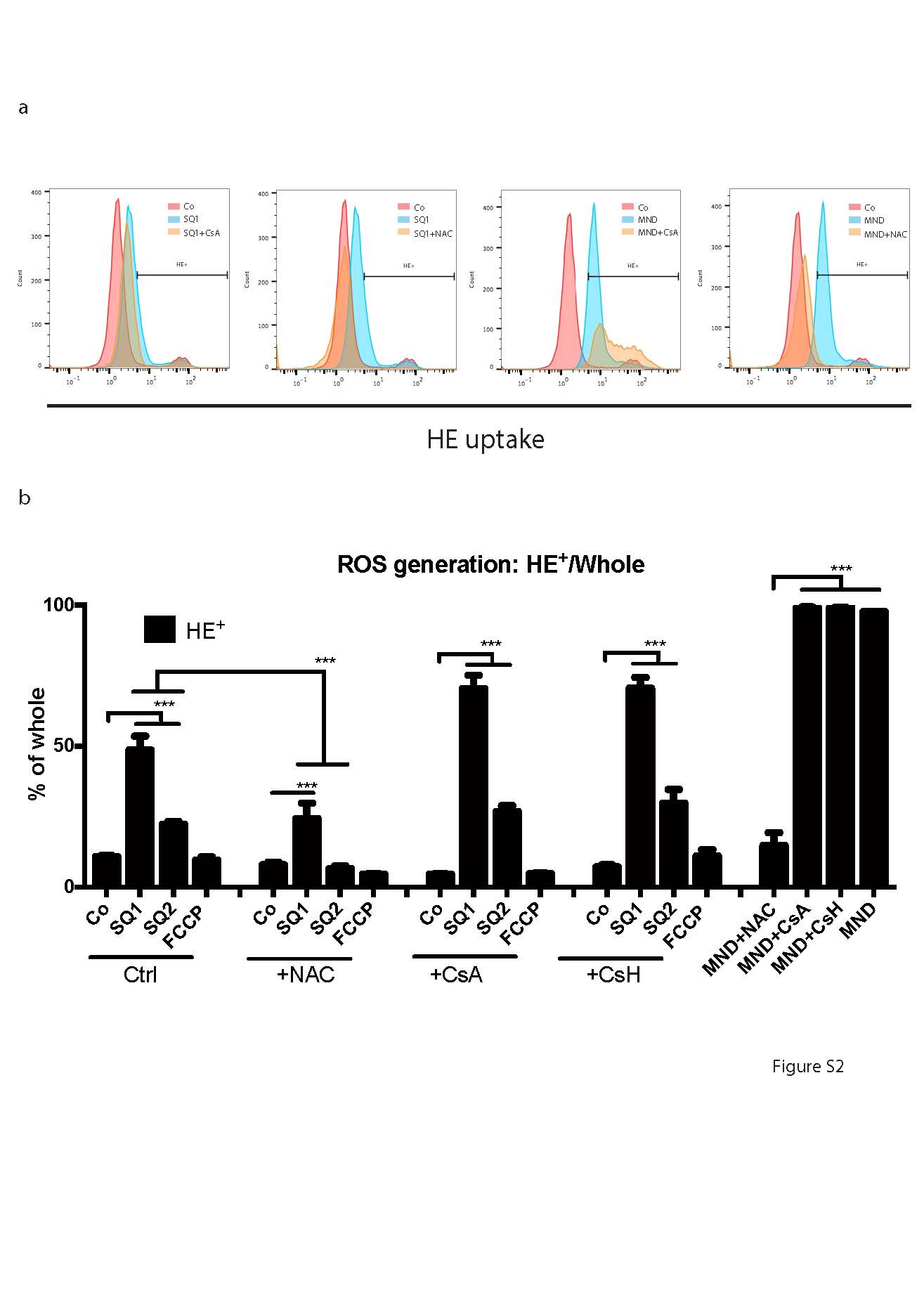

Supplement: Supplementary file 2 — Figure S2 [file 41419_2019_1474_MOESM2_ESM.jpg]

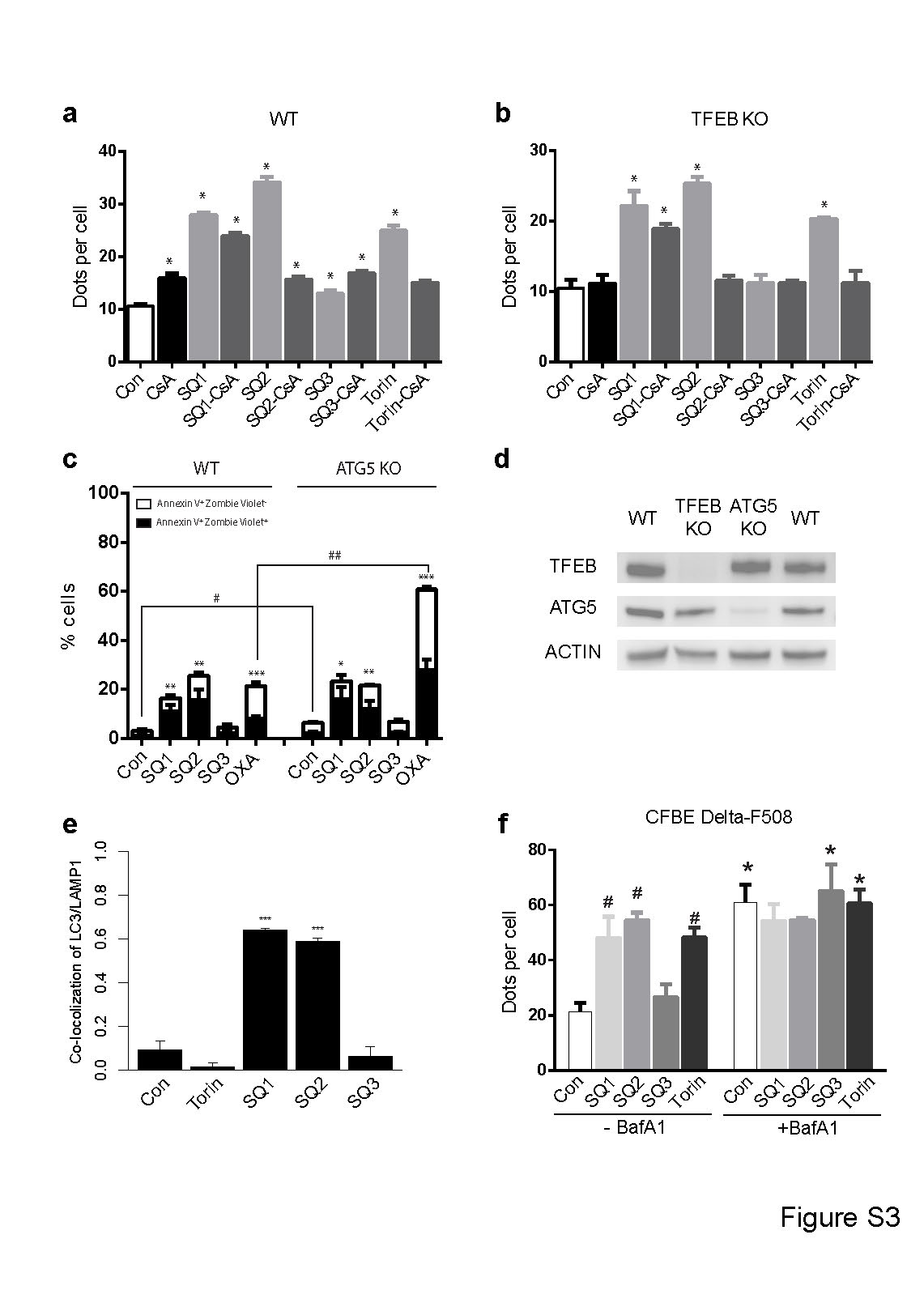

Supplement: Supplementary file 3 — Figure S3 [file 41419_2019_1474_MOESM3_ESM.jpg]
