# Supplementary material for: Squaramide-based synthetic chloride transporters activate TFEB but block autophagic flux
Source: Cell Death Dis. 2019 Mar 11;10(3):242. doi: 10.1038/s41419-019-1474-8 (PMC6411943; doi:10.1038/s41419-019-1474-8)
Supplement: Supplementary file 4 — Supplementary figure legends [file 41419_2019_1474_MOESM4_ESM.docx]

**Supplementary figure legend:**

**Figure S1. Quantitation of Lamp1 accumulation induced by the SQs.**

**a, b**) Representative images and statistical analysis of U2OS GFP-LC3 cells upon treatment with the indicated compounds for 6 hours followed by LAMP1 immunostaining. **c, d**) LAMP1 immunoblot and the correspondent densitometric analysis of protein extracts from U2OS cells treated with the synthetic chloride transporters for 6 hours. Data are expressed as means ± SEM of at least three independent experiments, *P<0.05; ***P<0.001.

**Figure S2. Effect of SQs on mitochondrial ROS.**

**a)** Representative histograms showing ROS detection by hydroethidine (HE) staining upon treatment of U2OSWT cells with **SQ1** (10 μM) and the positive control menadione (MND, 30 µM) for 6h. **b)** Bars represent the percentage of cell positive for ROS (that convert non-fluorescent HE into the fluorescent compounds ethidium). Data are expressed as means ± SEM of at least three independent experiments. Statistical analysis was done by comparing the average of the means of treated cells with the control cells (Co, Ctrl). ***P<0.001.

**Figure S3. Effect of SQs on the autophagic pathway.**

**a,b)** Side-by-side comparison of the effects of **SQ1** (10 μM), **SQ2** (10 μM), cyclosporin A (CsA, 1µM) and/or torin (300 nM) on GFP-LC3 dots formed in GFP-LC3 expressing WT U2OS cells (a) and TFEB KO U2OS cells (b), 6 hours after addition of the drugs. **c)** Cell death induction by SQ compounds (10 µM) compared to oxaliplatin (100 µM) in WT and ATG5 KO U2OS cells after 24 hours of culture. Statistical analysis was done by comparing the average of the means of treated cells with untreated control cells (Con). *P<0.05 ***P<0.001 or that of WT cells with KO cells. **d)** Confirmation of the genotype of WT, TFEB KO and ATG5 KO U2OS cells by immunoblot. **e)** Co-localization of GFP-LC3 with LAMP1 (determined by immunofluorescence staining) in U2OS WT cells treated with torin (100 nM) or SQ compounds after 6 hours of treatment. Statistical analysis was done by comparing the average of the means of treated cells with untreated control cells (Con). ***P<0.001. **f)** Effects of SQ compounds on CFBE Delta-F508 cells. CFBE cells stably expressing GFP-LC3. The cells were cultured for 6 hours with the indicated compounds, followed by quantitative assessment of GFP-LC3 dots. Statistical analysis was done by comparing the average of the means of treated cells with control cells (Con). #P<0.05 or by assessing the effect of bafilomycin A1 (BafA1) compared to cells cultured in the absence of BafA1 but otherwise similar conditions. *P<0.05.
